# Supplementary material for: Prevalence, treatment, and factors associated with cryptococcal meningitis post introduction of integrase inhibitors antiretroviral based regimens among People Living with HIV in Tanzania
Source: PLoS One. 2024 Feb 27;19(2):e0294940. doi: 10.1371/journal.pone.0294940 (PMC10898767; doi:10.1371/journal.pone.0294940)
Supplement: S1 Raw data — (DOCX) [file pone.0294940.s003.docx]

RAW DATA

*Table 1: Demographic characteristics of study participants (n=405)*

| **Statistics** | | |
| --- | --- | --- |
| Age(Years) | | |
| N | Valid | 405 |
|  | Missing | 0 |
| Mean | | 40.73 |
| Std. Error of Mean | | .678 |
| Median | | 43.00 |
| Std. Deviation | | 13.635 |
| Skewness | | -.247 |
| Std. Error of Skewness | | .121 |
| Range | | 76 |
| Minimum | | 2 |
| Maximum | | 78 |
| Percentiles | 25 | 31.00 |
|  | 50 | 43.00 |
|  | 75 | 50.00 |

| **Age category** | | | | | |
| --- | --- | --- | --- | --- | --- |
|  | | Frequency | Percent | Valid Percent | Cumulative Percent |
| Valid | <18 Years | 28 | 6.9 | 6.9 | 6.9 |
|  | 18-35 Years | 98 | 24.2 | 24.2 | 31.1 |
|  | 36-55 Years | 224 | 55.3 | 55.3 | 86.4 |
|  | >55 Years | 55 | 13.6 | 13.6 | 100.0 |
|  | Total | 405 | 100.0 | 100.0 |  |

| **Gender category** | | | | | |
| --- | --- | --- | --- | --- | --- |
|  | | Frequency | Percent | Valid Percent | Cumulative Percent |
| Valid | Male | 138 | 34.1 | 34.1 | 34.1 |
|  | Female | 267 | 65.9 | 65.9 | 100.0 |
|  | Total | 405 | 100.0 | 100.0 |  |

| **Occupation category** | | | | | |
| --- | --- | --- | --- | --- | --- |
|  | | Frequency | Percent | Valid Percent | Cumulative Percent |
| Valid | 1 | 88 | 21.7 | 21.7 | 21.7 |
|  | 2 | 152 | 37.5 | 37.5 | 59.3 |
|  | 3 | 165 | 40.7 | 40.7 | 100.0 |
|  | Total | 405 | 100.0 | 100.0 |  |

| **Marital status recategory** | | | | | |
| --- | --- | --- | --- | --- | --- |
|  | | Frequency | Percent | Valid Percent | Cumulative Percent |
| Valid | Married | 293 | 72.3 | 72.3 | 72.3 |
|  | Unmarried | 112 | 27.7 | 27.7 | 100.0 |
|  | Total | 405 | 100.0 | 100.0 |  |

| **ARTdefaulter category** | | | | | |
| --- | --- | --- | --- | --- | --- |
|  | | Frequency | Percent | Valid Percent | Cumulative Percent |
| Valid | yes | 37 | 9.1 | 9.1 | 9.1 |
|  | no | 368 | 90.9 | 90.9 | 100.0 |
|  | Total | 405 | 100.0 | 100.0 |  |

*Table 2: Prevalence cryptococcal meningitis, CD4 count at diagnosis, treatment approach and Outcome of treatment among participants (n=405)*

| **CrAg category** | | | | | |
| --- | --- | --- | --- | --- | --- |
|  | | Frequency | Percent | Valid Percent | Cumulative Percent |
| Valid | pos | 48 | 11.9 | 11.9 | 11.9 |
|  | neg | 357 | 88.1 | 88.1 | 100.0 |
|  | Total | 405 | 100.0 | 100.0 |  |

| **cd4atCrag+ category** | | | | | |
| --- | --- | --- | --- | --- | --- |
|  | | Frequency | Percent | Valid Percent | Cumulative Percent |
| Valid | <200 | 39 | 9.6 | 81.3 | 81.3 |
|  | ?200 | 9 | 2.2 | 18.8 | 100.0 |
|  | Total | 48 | 11.9 | 100.0 |  |
| Missing | 99 | 357 | 88.1 |  |  |
| Total | | 405 | 100.0 |  |  |

| **cragmed category** | | | | | |
| --- | --- | --- | --- | --- | --- |
|  | | Frequency | Percent | Valid Percent | Cumulative Percent |
| Valid | flu,5FU,amphotericin | 6 | 1.5 | 12.5 | 12.5 |
|  | fluconazole | 42 | 10.4 | 87.5 | 100.0 |
|  | Total | 48 | 11.9 | 100.0 |  |
| Missing | 99 | 357 | 88.1 |  |  |
| Total | | 405 | 100.0 |  |  |

| **outcome category** | | | | | |
| --- | --- | --- | --- | --- | --- |
|  | | Frequency | Percent | Valid Percent | Cumulative Percent |
| Valid | died | 4 | 1.0 | 8.9 | 8.9 |
|  | recovered | 18 | 4.4 | 40.0 | 48.9 |
|  | LTF | 17 | 4.2 | 37.8 | 86.7 |
|  | referred | 2 | .5 | 4.4 | 91.1 |
|  | on treatment | 4 | 1.0 | 8.9 | 100.0 |
|  | Total | 45 | 11.1 | 100.0 |  |
| Missing | 99 | 360 | 88.9 |  |  |
| Total | | 405 | 100.0 |  |  |

*Table 3: Showing Pearson chi-square test of the demographic characteristics and Cryptococcal meningitis status among participants (n = 405)*

| **Age category * CrAg category Crosstabulation** | | | | | |
| --- | --- | --- | --- | --- | --- |
|  | | | CrAg category | | Total |
|  |  |  | pos | neg |  |
| Age category | <18 Years | Count | 0 | 28 | 28 |
|  |  | % within Age category | 0.0% | 100.0% | 100.0% |
|  | 18-35 Years | Count | 17 | 81 | 98 |
|  |  | % within Age category | 17.3% | 82.7% | 100.0% |
|  | 36-55 Years | Count | 25 | 199 | 224 |
|  |  | % within Age category | 11.2% | 88.8% | 100.0% |
|  | >55 Years | Count | 6 | 49 | 55 |
|  |  | % within Age category | 10.9% | 89.1% | 100.0% |
| Total | | Count | 48 | 357 | 405 |
|  |  | % within Age category | 11.9% | 88.1% | 100.0% |

| **Chi-Square Tests** | | | |
| --- | --- | --- | --- |
|  | Value | df | Asymp. Sig. (2-sided) |
| Pearson Chi-Square | 6.746^a^ | 3 | .080 |
| Likelihood Ratio | 9.741 | 3 | .021 |
| N of Valid Cases | 405 |  |  |
| a. 1 cells (12.5%) have expected count less than 5. The minimum expected count is 3.32. | | | |

| **Gender category * CrAg category Crosstabulation** | | | | | |
| --- | --- | --- | --- | --- | --- |
|  | | | CrAg category | | Total |
|  |  |  | pos | neg |  |
| Gender category | Male | Count | 23 | 115 | 138 |
|  |  | % within Gender category | 16.7% | 83.3% | 100.0% |
|  | Female | Count | 25 | 242 | 267 |
|  |  | % within Gender category | 9.4% | 90.6% | 100.0% |
| Total | | Count | 48 | 357 | 405 |
|  |  | % within Gender category | 11.9% | 88.1% | 100.0% |

| **Chi-Square Tests** | | | | | |
| --- | --- | --- | --- | --- | --- |
|  | Value | df | Asymp. Sig. (2-sided) | Exact Sig. (2-sided) | Exact Sig. (1-sided) |
| Pearson Chi-Square | 4.645^a^ | 1 | .031 |  |  |
| Continuity Correction^b^ | 3.972 | 1 | .046 |  |  |
| Likelihood Ratio | 4.454 | 1 | .035 |  |  |
| Fisher's Exact Test |  |  |  | .036 | .025 |
| N of Valid Cases | 405 |  |  |  |  |
| a. 0 cells (0.0%) have expected count less than 5. The minimum expected count is 16.36. | | | | | |
| b. Computed only for a 2x2 table | | | | | |

| **Occupation category * CrAg category Crosstabulation** | | | | | |
| --- | --- | --- | --- | --- | --- |
|  | | | CrAg category | | Total |
|  |  |  | pos | neg |  |
| Occupation category | 1 | Count | 8 | 80 | 88 |
|  |  | % within Occupation category | 9.1% | 90.9% | 100.0% |
|  | 2 | Count | 20 | 132 | 152 |
|  |  | % within Occupation category | 13.2% | 86.8% | 100.0% |
|  | 3 | Count | 20 | 145 | 165 |
|  |  | % within Occupation category | 12.1% | 87.9% | 100.0% |
| Total | | Count | 48 | 357 | 405 |
|  |  | % within Occupation category | 11.9% | 88.1% | 100.0% |

| **Chi-Square Tests** | | | |
| --- | --- | --- | --- |
|  | Value | df | Asymp. Sig. (2-sided) |
| Pearson Chi-Square | .902^a^ | 2 | .637 |
| Likelihood Ratio | .943 | 2 | .624 |
| N of Valid Cases | 405 |  |  |
| a. 0 cells (0.0%) have expected count less than 5. The minimum expected count is 10.43. | | | |

| **Marital status recategory * CrAg category Crosstabulation** | | | | | |
| --- | --- | --- | --- | --- | --- |
|  | | | CrAg category | | Total |
|  |  |  | pos | neg |  |
| Marital status recategory | Married | Count | 24 | 269 | 293 |
|  |  | % within Marital status recategory | 8.2% | 91.8% | 100.0% |
|  | Unmarried | Count | 24 | 88 | 112 |
|  |  | % within Marital status recategory | 21.4% | 78.6% | 100.0% |
| Total | | Count | 48 | 357 | 405 |
|  |  | % within Marital status recategory | 11.9% | 88.1% | 100.0% |

| **Chi-Square Tests** | | | | | |
| --- | --- | --- | --- | --- | --- |
|  | Value | df | Asymp. Sig. (2-sided) | Exact Sig. (2-sided) | Exact Sig. (1-sided) |
| Pearson Chi-Square | 13.591^a^ | 1 | .000 |  |  |
| Continuity Correction^b^ | 12.353 | 1 | .000 |  |  |
| Likelihood Ratio | 12.344 | 1 | .000 |  |  |
| Fisher's Exact Test |  |  |  | .000 | .000 |
| Linear-by-Linear Association | 13.557 | 1 | .000 |  |  |
| N of Valid Cases | 405 |  |  |  |  |
| a. 0 cells (0.0%) have expected count less than 5. The minimum expected count is 13.27. | | | | | |
| b. Computed only for a 2x2 table | | | | | |

| **ARTdefaulter category * CrAg category Crosstabulation** | | | | | |
| --- | --- | --- | --- | --- | --- |
|  | | | CrAg category | | Total |
|  |  |  | pos | neg |  |
| ARTdefaulter category | yes | Count | 37 | 0 | 37 |
|  |  | % within ARTdefaulter category | 100.0% | 0.0% | 100.0% |
|  | no | Count | 11 | 357 | 368 |
|  |  | % within ARTdefaulter category | 3.0% | 97.0% | 100.0% |
| Total | | Count | 48 | 357 | 405 |
|  |  | % within ARTdefaulter category | 11.9% | 88.1% | 100.0% |

| **Chi-Square Tests** | | | | | |
| --- | --- | --- | --- | --- | --- |
|  | Value | df | Asymp. Sig. (2-sided) | Exact Sig. (2-sided) | Exact Sig. (1-sided) |
| Pearson Chi-Square | 302.856^a^ | 1 | .000 |  |  |
| Continuity Correction^b^ | 293.641 | 1 | .000 |  |  |
| Likelihood Ratio | 195.918 | 1 | .000 |  |  |
| Fisher's Exact Test |  |  |  | .000 | .000 |
| N of Valid Cases | 405 |  |  |  |  |
| a. 1 cells (25.0%) have expected count less than 5. The minimum expected count is 4.39. | | | | | |
| b. Computed only for a 2x2 table | | | | | |

**Table 4:** *Showing Pearson chi-square test of the demographic characteristics and Cryptococcal meningitis status among participants (n=45)*

N: B. LTF means Loss to Follow Up

| **Age category * outcome category Crosstabulation** | | | | | | | | |
| --- | --- | --- | --- | --- | --- | --- | --- | --- |
|  | | | outcome category | | | | | Total |
|  |  |  | died | recovered | LTF | referred | on treatment |  |
| Age  category | 18-35 Years | Count | 0 | 7 | 7 | 1 | 0 | 15 |
|  |  | % within Age category | 0.0% | 46.7% | 46.7% | 6.7% | 0.0% | 100.0% |
|  | 36-55 Years | Count | 2 | 10 | 8 | 1 | 3 | 24 |
|  |  | % within Age category | 8.3% | 41.7% | 33.3% | 4.2% | 12.5% | 100.0% |
|  | >55 Years | Count | 2 | 1 | 2 | 0 | 1 | 6 |
|  |  | % within Age category | 33.3% | 16.7% | 33.3% | 0.0% | 16.7% | 100.0% |
| Total | | Count | 4 | 18 | 17 | 2 | 4 | 45 |
|  |  | % within Age category | 8.9% | 40.0% | 37.8% | 4.4% | 8.9% | 100.0% |

| **Chi-Square Tests** | | | |
| --- | --- | --- | --- |
|  | Value | df | Asymp. Sig. (2-sided) |
| Pearson Chi-Square | 9.377^a^ | 8 | .312 |
| Likelihood Ratio | 10.692 | 8 | .220 |
| N of Valid Cases | 45 |  |  |
| a. 11 cells (73.3%) have expected count less than 5. The minimum expected count is .27. | | | |

| **Gender category * outcome category Crosstabulation** | | | | | | | | |
| --- | --- | --- | --- | --- | --- | --- | --- | --- |
|  | | | outcome category | | | | | Total |
|  |  |  | died | recovered | LTF | referred | on treatment |  |
| Gender  category | Male | Count | 3 | 9 | 7 | 1 | 1 | 21 |
|  |  | % within Gender category | 14.3% | 42.9% | 33.3% | 4.8% | 4.8% | 100.0% |
|  | Female | Count | 1 | 9 | 10 | 1 | 3 | 24 |
|  |  | % within Gender category | 4.2% | 37.5% | 41.7% | 4.2% | 12.5% | 100.0% |
| Total | | Count | 4 | 18 | 17 | 2 | 4 | 45 |
|  |  | % within Gender category | 8.9% | 40.0% | 37.8% | 4.4% | 8.9% | 100.0% |

| **Chi-Square Tests** | | | |
| --- | --- | --- | --- |
|  | Value | df | Asymp. Sig. (2-sided) |
| Pearson Chi-Square | 2.340^a^ | 4 | .674 |
| Likelihood Ratio | 2.425 | 4 | .658 |
| N of Valid Cases | 45 |  |  |
| a. 6 cells (60.0%) have expected count less than 5. The minimum expected count is .93. | | | |

| **Occupation category * outcome category Crosstabulation** | | | | | | | | |
| --- | --- | --- | --- | --- | --- | --- | --- | --- |
|  | | | outcome category | | | | | Total |
|  |  |  | died | recovered | LTF | referred | on treatment |  |
| Occupation category | 1 | Count | 0 | 2 | 4 | 1 | 0 | 7 |
|  |  | % within Occupation category | 0.0% | 28.6% | 57.1% | 14.3% | 0.0% | 100.0% |
|  | 2 | Count | 1 | 7 | 10 | 0 | 2 | 20 |
|  |  | % within Occupation category | 5.0% | 35.0% | 50.0% | 0.0% | 10.0% | 100.0% |
|  | 3 | Count | 3 | 9 | 3 | 1 | 2 | 18 |
|  |  | % within Occupation category | 16.7% | 50.0% | 16.7% | 5.6% | 11.1% | 100.0% |
| Total | | Count | 4 | 18 | 17 | 2 | 4 | 45 |
|  |  | % within Occupation category | 8.9% | 40.0% | 37.8% | 4.4% | 8.9% | 100.0% |

| **Chi-Square Tests** | | | |
| --- | --- | --- | --- |
|  | Value | df | Asymp. Sig. (2-sided) |
| Pearson Chi-Square | 9.815^a^ | 8 | .278 |
| Likelihood Ratio | 11.574 | 8 | .171 |
| N of Valid Cases | 45 |  |  |
| a. 11 cells (73.3%) have expected count less than 5. The minimum expected count is .31. | | | |

| **Marital status recategory * outcome category Crosstabulation** | | | | | | | | | | | | | | | | | | | | |
| --- | --- | --- | --- | --- | --- | --- | --- | --- | --- | --- | --- | --- | --- | --- | --- | --- | --- | --- | --- | --- |
|  | | | | | | | outcome category | | | | | | | | | | | | Total | |
|  |  |  |  |  |  |  | died | | | recovered | | | LTF | | referred | | on treatment | |  |  |
| Marital status recategory | Married | | Count | | | 3 | | | 11 | | | 7 | | 0 | | 1 | | 22 | |  |
|  |  |  | % within Marital status recategory | | | 13.6% | | | 50.0% | | | 31.8% | | 0.0% | | 4.5% | | 100.0% | |  |
|  | Unmarried | | Count | | | 1 | | | 7 | | | 10 | | 2 | | 3 | | 23 | |  |
|  |  |  | % within Marital status recategory | | | 4.3% | | | 30.4% | | | 43.5% | | 8.7% | | 13.0% | | 100.0% | |  |
| Total | | | | Count | | | 4 | | | 18 | | | 17 | | 2 | | 4 | | 45 | |
|  |  |  |  | % within Marital status recategory | | | 8.9% | | | 40.0% | | | 37.8% | | 4.4% | | 8.9% | | 100.0% | |
| **Chi-Square Tests** | | | | | | | | | | |  |  |  |  |  |  |  |  |  |  |
|  | | Value | | | df | | | Asymp. Sig. (2-sided) | | |  |  |  |  |  |  |  |  |  |  |
| Pearson Chi-Square | | 5.399^a^ | | | 4 | | | .249 | | |  |  |  |  |  |  |  |  |  |  |
| Likelihood Ratio | | 6.272 | | | 4 | | | .180 | | |  |  |  |  |  |  |  |  |  |  |
| N of Valid Cases | | 45 | | |  | | |  | | |  |  |  |  |  |  |  |  |  |  |
| a. 6 cells (60.0%) have expected count less than 5. The minimum expected count is .98. | | | | | | | | | | |  |  |  |  |  |  |  |  |  |  |

| **ARTdefaulter category * outcome category Crosstabulation** | | | | | | | | |
| --- | --- | --- | --- | --- | --- | --- | --- | --- |
|  | | | outcome category | | | | | Total |
|  |  |  | died | recovered | LTF | referred | on treatment |  |
| ARTdefaulter category | yes | Count | 3 | 14 | 12 | 1 | 4 | 34 |
|  |  | % within ARTdefaulter category | 8.8% | 41.2% | 35.3% | 2.9% | 11.8% | 100.0% |
|  | no | Count | 1 | 4 | 5 | 1 | 0 | 11 |
|  |  | % within ARTdefaulter category | 9.1% | 36.4% | 45.5% | 9.1% | 0.0% | 100.0% |
| Total | | Count | 4 | 18 | 17 | 2 | 4 | 45 |
|  |  | % within ARTdefaulter category | 8.9% | 40.0% | 37.8% | 4.4% | 8.9% | 100.0% |

| **Chi-Square Tests** | | | |
| --- | --- | --- | --- |
|  | Value | df | Asymp. Sig. (2-sided) |
| Pearson Chi-Square | 2.277^a^ | 4 | .685 |
| Likelihood Ratio | 3.116 | 4 | .539 |
| N of Valid Cases | 45 |  |  |
| a. 8 cells (80.0%) have expected count less than 5. The minimum expected count is .49. | | | |

| **cd4atCrag+ category * outcome category Crosstabulation** | | | | | | | | |
| --- | --- | --- | --- | --- | --- | --- | --- | --- |
|  | | | outcome category | | | | | Total |
|  |  |  | died | recovered | LTF | referred | on treatment |  |
| cd4atCrag+ category | <200 | Count | 4 | 12 | 15 | 2 | 3 | 36 |
|  |  | % within cd4atCrag+ category | 11.1% | 33.3% | 41.7% | 5.6% | 8.3% | 100.0% |
|  | ?200 | Count | 0 | 6 | 2 | 0 | 1 | 9 |
|  |  | % within cd4atCrag+ category | 0.0% | 66.7% | 22.2% | 0.0% | 11.1% | 100.0% |
| Total | | Count | 4 | 18 | 17 | 2 | 4 | 45 |
|  |  | % within cd4atCrag+ category | 8.9% | 40.0% | 37.8% | 4.4% | 8.9% | 100.0% |

| **Chi-Square Tests** | | | |
| --- | --- | --- | --- |
|  | Value | df | Asymp. Sig. (2-sided) |
| Pearson Chi-Square | 4.283^a^ | 4 | .369 |
| Likelihood Ratio | 5.308 | 4 | .257 |
| N of Valid Cases | 45 |  |  |
| a. 8 cells (80.0%) have expected count less than 5. The minimum expected count is .40. | | | |

| **cragmed category * outcome category Crosstabulation** | | | | | | | | |
| --- | --- | --- | --- | --- | --- | --- | --- | --- |
|  | | | outcome category | | | | | Total |
|  |  |  | died | recovered | LTF | referred | on treatment |  |
| cragmed category | flu,5FU,amphotericin | Count | 0 | 3 | 0 | 0 | 2 | 5 |
|  |  | % within cragmed category | 0.0% | 60.0% | 0.0% | 0.0% | 40.0% | 100.0% |
|  | fluconazole | Count | 4 | 15 | 17 | 2 | 2 | 40 |
|  |  | % within cragmed category | 10.0% | 37.5% | 42.5% | 5.0% | 5.0% | 100.0% |
| Total | | Count | 4 | 18 | 17 | 2 | 4 | 45 |
|  |  | % within cragmed category | 8.9% | 40.0% | 37.8% | 4.4% | 8.9% | 100.0% |

| **Chi-Square Tests** | | | |
| --- | --- | --- | --- |
|  | Value | df | Asymp. Sig. (2-sided) |
| Pearson Chi-Square | 9.563^a^ | 4 | .048 |
| Likelihood Ratio | 9.630 | 4 | .047 |
| N of Valid Cases | 45 |  |  |
| a. 8 cells (80.0%) have expected count less than 5. The minimum expected count is .22. | | | |
